# Single-molecule 3D imaging of HIV cellular entry by liquid-phase electron tomography

**DOI:** 10.21203/rs.3.rs-1298112/v1

**Published:** 2022-02-04

**Authors:** Lingli Kong, Jianfang Liu, Meng Zhang, Zhuoyang Lu, Amy Ren, Jiankang Liu, Jinping Li, Gang (Gary) Ren

**Affiliations:** Lawrence Berkeley National Laboratory; Lawrence Berkeley National Laboratory; Lawrence Berkeley National Laboratory; Lawrence Berkeley National Laboratory; Lawrence Berkeley National Laboratory; Xi'an Jiaotong University; Lawrence Berkeley National Laboratory; Lawrence Berkeley National Laboratory

## Abstract

Enveloped viruses, including human immunodeficiency virus (HIV) and SARS-CoV-2, target cells through membrane fusion process. The detailed understanding of the process is sought after for vaccine development but remains elusive due to current technique limitations for direct three-dimensional (3D) imaging of an individual virus during its viral entry. Recently, we developed a simple specimen preparation method for real-time imaging of metal dynamic liquid-vaper interface at nanometer resolution by transmission electron microscopy (TEM). Here, we extended this method to study biology sample through snapshot 3D structure of a single HIV (pseudo-typed with the envelope glycoprotein of vesicular stomatitis virus, VSV-G) at its intermediate stage of viral entry to HeLa cells in a liquid-phase environment. By individual-particle electron tomography (IPET), we found the viral surface release excess lipids with unbound viral spike proteins forming ~50-nm nanoparticles instead of merging cell membrane. Moreover, the spherical-shape shell formed by matrix proteins underneath the viral envelope does not disassemble into a cone shape right after fusion. The snapshot 3D imaging of a single virus provides us a direct structure-based understanding of the viral entry mechanism, which can be used to examine other viruses to support the development of vaccines combatting the current ongoing pandemic.

## Introduction

Human immunodeficiency virus (HIV), syndrome coronavirus 2 (SARS-CoV-2), and influenza A virus are all belong to the family of enveloped viruses, which contain a lipid bilayer membrane as an envelope surrounding the virus particle. Although enveloped virus entry into target cells are very diverse in their pathways, they all share many common patterns,^1,2^ such as beginning with attachment to cell-surface receptors, followed by fusion of the virus membrane to the cell membrane, and ending with penetration and delivery of the viral genome to the cell cytoplasm. HIV, as a type of lentiviruses, also belong to the family of retroviruses,^3^ which can deliver their viral genomic RNA into the mammalian cells for replication.^4^ Due to technique limitations in observation of this dynamic process of viral entry at the nanometer resolution, the rapid intermediate fusion process at unpredictable locations of viral landings has rarely been observed.

Electron tomography (ET) provides an ideal approach for high-resolution 3D imaging of an individual biological object^5-9^ through a series of tilt views, regardless of its conformations or the number of surrounding molecules. However, EM techniques still face challenges in catching the intermediates of viral entry in 3D, especially in a liquid-phase environment. The cryo-ET method requires frozen-hydrated cells to provide a low image contrast of virus near the cell surface^13-15^. For high contrast imaging, it is generally required to slice native cells with a physical knife or electron beam for more transmission through. However, slicing by physical microtome knife could damage the cell surface structure, such as by contamination of endosome components,^16^ while slicing by focused ion beam (FIB) could heat and devitrify the surface of cell.^17,18^ Alternatively, for high contrast imaging, cells can be also chemically fixed and plastic imbedded, such as the progressive lowering of temperature (PLT)^19^, in which the cells could be damaged by artifacts including distorted cellular membranes or organelles and the loss of material, making the cell appear less dense ^20^.

Probing the detailed processes of membrane organization and HIV viral penetration remains challenging, especially in a liquid-phase environment.^21^ Liquid-phase TEM has been developed near a century ^24,25^, in which the electron beam needs to pass through a thin layer of a liquid solution hermetically encapsulated between two thin layers of electron-permeable films.^26,27^ The early development included using collodion films ^27^ as barriers between the liquid and gas phases ^26,28-30^ for examination of adequate structure of biology samples ^29^. In latest decade, nano-fabricated silicon-nitride film ^31-35^ has been widely used as barriers for high-resolution and real-time TEM imaging of nano-crystal interactions and chemical reactions in liquid-phase environments ^36-39^. For whole cell imaging, scanning transmission electron microscopy (STEM) has been used for nanometer resolution imaging ^37,40,41^. Although silicon-nitride film provide a robust barrier to encapsulate liquid-phase sample under high-vacuum of TEM, the encapsulated specimen must be mounted on the tip of specially designed holders,^33^ which generated an additional barrier for regular researchers to easily utilize this technique.

To image the sample in liquid-phase state easily, we recently reported a simple method ^42^ in which two plastic Formvar films were used to encapsulate a liquid sample. By this method, we imaged for first-time the dynamic structure of the liquid-vapor interface at ~1 nm resolution for over one hour period ^42^. Here, we implemented this simple designed method to study the viral entry process via imaging the intermediate states of HIV viral entry to HeLa cells in a liquid-phase state. The HIV used here is a pseudo-typed HIV virus with VSV-G that can infect HeLa cells. This virus was designed as a replication-incompetent HIV to infect cells for desired protein production^43^, via expression of nonfunctional single-stranded RNA (siRNA). To achieve a 3D density map of the virus at its intermediate stages of viral entry, we used reported individual-particle electron tomography (IPET) method^9^, an approach for 3D structural determination of a single protein particle to quantitatively characterize the structural dynamics of flexible biomolecules, such as DNA nanoparticles ^6,10^ and antibodies ^8,11,12^. This sandwiching approach can avoid the potential contamination from the endosome components or distortion from slicing and provided an excellent image contrast compared to cryo-EM as well as more structural details than the silicon-nitride film encapsulated liquid-phase sample.

## Results

### TEM imaging of proteins in liquid-phase solution

**Figure 1:**
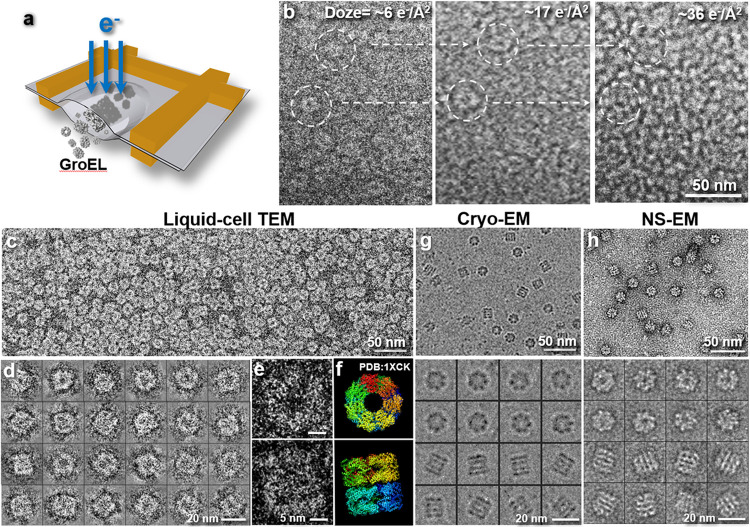
Liquid-phase TEM images of GroEL in TBS buffer compared to cryo-EM and NS images. **a,** schematics of the assembly of liquid-phase TEM grids with encapsulated GroEL particles in TBS buffer. **b,** electron radiation damage shows a “bubbling” phenomenon caused by a series of cumulative illumination doses (from left to right) of 1.0 to 36.2 e^−^/Å^2^. **c,** survey view (high-pass filtered) of GroEL particles imaged under optimized illumination conditions shows a uniform distribution. **d,** representative particles show the structures of GroEL particles in two distinguishable orientations. **e,** a representative zoomed-in image of two particles in different orientations shows structural details similar to **f,** the crystal structure (PDB 1XCK) viewed along its *C_7_* symmetric axis and *C_2_* symmetric axis. **g,** comparison to the cryo-EM images of GroEL, Copyright Vossman, CC BY-SA 4.0, via Wikimedia Commons. **h,** comparing to the NS image of the same sample.

For a proof-of-concept, the liquid-phase TEM specimen preparation method used for material science research can be effectively used for examining a biological sample. We first examined the protein GroEL, a standard sample that is commonly used for testing new approaches in the EM field. GroEL protein is a chaperone protein with a molecular mass of 800 kDa and a cylinder-shaped D_7_ symmetric structure with dimensions of ~13.5 nm x ~14.5 nm. A small amount (~0.2 μl at ~0.1 mg/ml) of label-free, stain-free, native GroEL protein in buffer was sandwiched between two formvar films and examined at room temperature (Fig. 1a). Survey micrographs showed donut-shaped particles with a diameter of ~14 nm. However, the shapes of the particles can be easily damaged by the cumulative effect the electron beam (Fig. 1b, and Extended Data Fig. 1). This damage includes sample deformation and bubbling caused by ionizing radiation that induces radiolysis of liquid molecules and leads to evaporation and the formation of gaseous byproducts ^44^. The bubbling phenomenon induced by electron beam is often observed in cryo-EM and liquid-phase TEM ^52,53^, but not in negative-staining (NS) or dried samples, suggesting that the cryo-electron microscopy (cryo-EM) sample is wet. Under the optimized illumination conditions, the high-resolution images of GroEL in liquid buffer reveal rich detailed features, such as the domain structures (Fig. 1c-e), which are consistent with those from the crystal structure (PDB entry: 1XCK,^45^ Fig. 1f) and cryo-EM images (Fig. 1g) but richer than those from NS (Fig. 1h). Notably, the biomolecular image contrast is same as that obtained by NS (Fig. 1h) and the silicon-nitride film encapsulated liquidphase TEM sample, such as the simian rotavirus double-layered particles (DLPs) ^24^. However, the contrast is inverted to that obtained by cryo-EM (Fig. 1g). Several reasons may cause that the increased salt concentration due to surrounding water partially evaporation during specimen preparation. A small amount of water evaporation from a tiny amount of solution (~0.2 μl) will significantly increase the salt concentration, resulting the electron scattering capability by the surrounding solution to be higher than from proteins. The heavier elements or ions in the salts have a much higher scattering than the light elements in the proteins ^46,47^. A higher solvent concentration from native buffer is usually not fatal to the protein structure, which often occurred in protein crystallization and chromatography^48,49^. During protein crystallization, the surrounding layers of water maintaining the protein structure were intended to evaporate gradually^50^. The dark shadow surrounding the particles shown in NS images, but not shown in liquid TEM or cryo-EM, suggests that the high solvent content is evenly distributed in the background solution instead of accumulating in the protein particle surroundings. Our liquid TEM images showed a higher resolution structural details than that from NS or silicon-nitride film based liquid TEM, ^24^ suggesting that this simple method is suitable for examining proteins, virus and cells.

### Liquid-phase TEM images of HeLa cells and HIV infected HeLa cells

**Figure 2:**
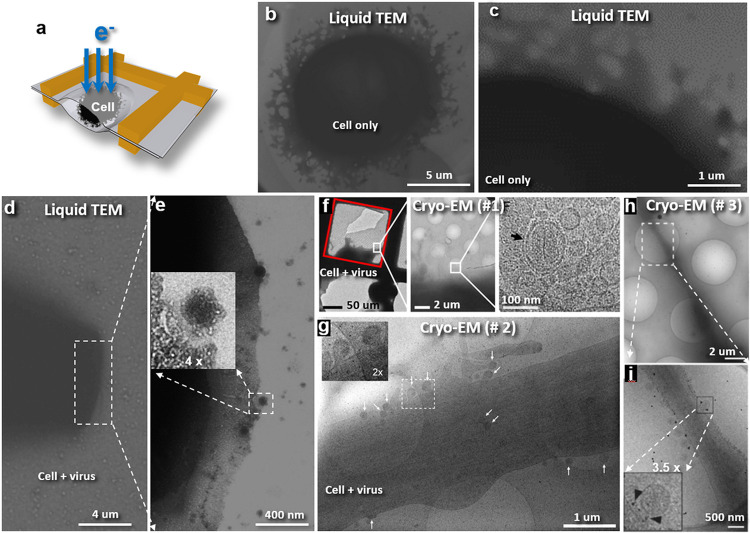
Liquid-phase TEM images of cell and virus compared to cryo-EM images. **a,** Schematics of assembling of liquid-phase TEM specimens that the samples are encapsulated by sandwiched between two Formvar films. **b** Representative liquid TEM images of a HeLa cell, shown in soft irregulated shape. **c,** The image of the cell edge under relatively strong electron beam. **d,** A rectangle-shaped HeLa cell infected by HIVs, in which the rectangle area is zoomed-in to show, **e,** The cell boundary and surrounding virus (after high-pass filtered image). **f,** The first comparison cryo-EM images of viral infected cell published ^13^, *i.e.* the HeLa cells at stage of HIV-1 infection. The HIV-1 is a pseudo-typed virion with the envelope glycoprotein, VSV-G, and containing GFP fused to HIV-1 Vpr (reproduced images with the permit of copyright). **g,** The second comparison cryo-EM images of viral infected cell published ^14^, *i.e.* the assembled HIV-1 was budding from the surface of U-373 MG cell (reproduced images with the permit of copyright **h** and **i,** The third comparison cryo-EM images of viral infected cell published ^15^, *i.e.* human respiratory syncytial virus (hRSV) infected HeLa cells with the hRSV F glycoprotein immunolabeled (reproduced images with the permit of copyright

HeLa cells alone and the HIV infected HeLa cells in growth medium were then encapsulated and examined by TEM respectively (Fig. 2a). The survey micrographs of cells exhibited soft-irregular shaped HeLa cells (Fig. 2b) consistent to the living HeLa cells imaged by super-resolution fluence light microscopy ^51^. Moreover, bubbling induced by electron beam can be often observed (Extended Data Fig. 2a and b) as that of other liquid-phase TEM ^52,53^. Plus, the radiation sensitivity of the cell membrane (Fig. 2c and Extended Data Fig. 2c) further confirmed that the specimen was wet.

The survey micrographs of HIV infected cells confirmed the soft-irregular shapes of HeLa cells. The irregular shape of cell membrane was often overlapped with each other, making the virus difficult to be clearly identified. However, on some cell surface, the roundish viruses were clearly visible on the cell surface (Fig. 2d and e). The virus images have a much stronger contrast than that from the cryo-EM (Fig. 2f-i), suggesting a unique advancement of this method in studying the virus and cell.

**Figure 3:**
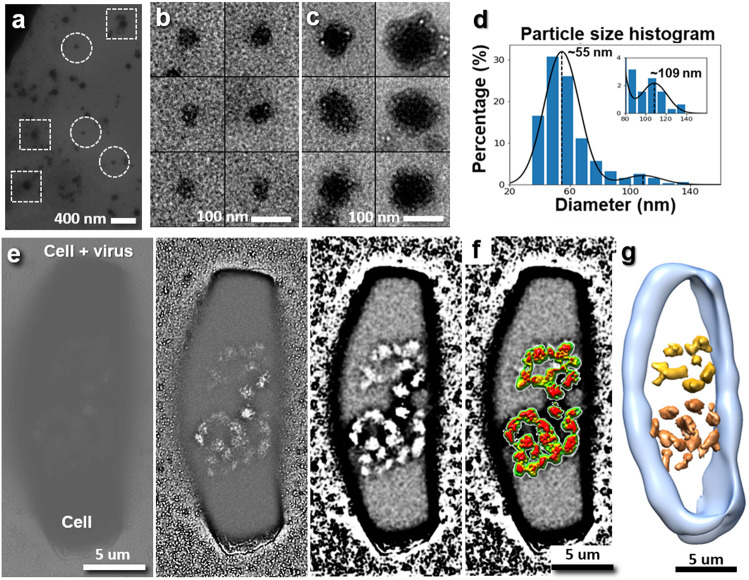
Liquid-phase TEM images of HeLa cells in liquid growth medium. **a,** A representative image of the surrounding background in a sample of a virus-infected HeLa cell, containing small nanoparticles and large virus-like particles indicated in circles and squares, respectively. **b and c,** 6 representative images of small nanoparticles and large virus-like particles, respectively. **d,** Histogram of a total of 315 particle diameters shows the major peak at ~55 nm and the second peak at ~109. **e and f,** Detailed internal cell structure shown by high-contrast image superimposed with its topography image of the center density portion. **g,** IPET 3D reconstruction from series of tilting images showing the architecture of this cell by superimposing positive and negative densities into one 3D map at 227 nm resolution.

Surrounding the cells, there are many particles in diameters from ~20 nm to ~200 nm (Fig. 3a-c). The statistical analysis showed that smaller particles (<90 nm, Fig. 3b) have a peak population with a diameter of ~55 nm (Fig. 3d), while larger particles (Fig. 3c) have a peak population with a diameter of ~109 nm (Fig. 3d). Considering HIV usually has a spherical shape with varying diameters ranging from 64 to 170 nm ^54,55^, the large particles are similar in size to HIV^11,12^, and consistent with the observation of HIV by cryo-EM (Fig. 2f-i).

By zooming in a rectangular shaped HeLa cell with smooth cell membrane (in dimension of ~17 μm × ~6 μm, Fig. 3e), we found two chain-shaped densities present near the center (Fig. 3f). ET tilt series of images acquired under a low magnification (~1,000 ×) showed two distal ends having concave surface shape between two films in distance of ~ 1 um (Fig. 3g, Extended Data Fig. 3g and Supplementary Video S1). The IPET 3D reconstruction of the cell confirmed two chain-shaped densities with similar size and shape near the cell center (Fig. 3g, and Supplementary Video S1). The cell shape and two “copies” of center densities suggest this cell is in the middle of cell division, *i.e.*, extending or lengthening itself to split its chromosomes into two halves.

### The intermediates of viral entry into cell

**Figure 4:**
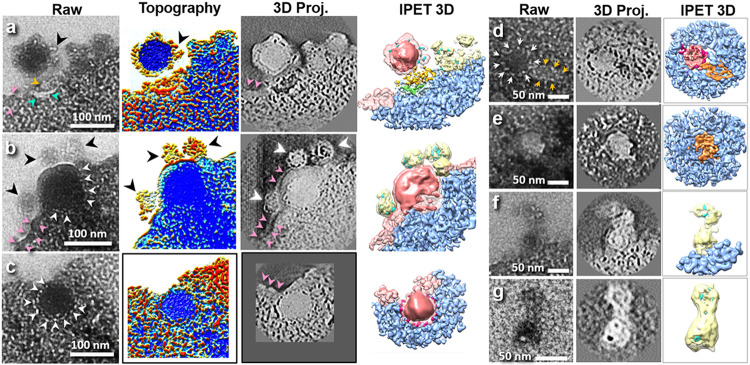
liquid-phase TEM images and IPET 3D reconstructions of HIV and related particles. **a,** Landing stage: zoomed-in raw micrograph, its colorized topography, IPET 3D center slice and superimposed 3D density map (a combination of positive and negative density maps at ~7.2 nm resolution). Cell surface membrane becomes concave in response to viral attachment. Yellow and cyan arrows indicate two types of the accumulated proteins involved in the virus-cell interaction. The black arrow points to viral spikes that are released from the viral surface and move toward a small nanoparticle, while the pink arrows indicate the abnormally thick membrane. **b,** Penetrating stage: zoomed-in raw micrograph, its colorized topography, IPET 3D center slice and superimposed 3D density map (at ~7.4 nm resolution) show the virus half imbedded inside the cell membrane, in which its surface spike proteins disappear from the outside half of a virus, where three ~50-nm small nanoparticles containing spike-size densities appeared on this surface (indicated by three black arrows). The surrounding densities distinguish the roundish shape of the half virus inside of cell (white arrows). Abnormal thickness of the membrane boundary is indicated by pink arrows. **c,** Imbedded stage: zoomed-in raw micrograph, its colorized topography, IPET 3D center slice and superimposed 3D density map (at ~6.5 nm resolution) show a virus that has just penetrated the cell surface. The arrows indicate that the particles exhibited a roundish virus shape underneath the membrane; the pink arrows indicate the abnormal thickness of the membrane boundary. **d,** A representative endocytosed particle (indicated by white arrows) attached to a polygon-shaped object (indicated by orange arrows). The 3D map resolution is ~6.4 nm. **e,** A polygon-shaped particle inside the cell. The 3D map resolution is ~6.6 nm. **(f and g)** Two representative extracellular nanoparticles surrounding the cell. The resolutions of the 3D maps are ~7.5 nm and ~9.1 nm respectively.

The dividing cell provided an ideal thin cell membrane with a smooth edge at its distal ends, which was easy to distinguish the viruses from the cell surface (Fig. 2d and e, and Supplementary Video 2). Taking advantage of these properties, we observed the several intermediate stages of viral entry (Fig. 4a-c) as following: i) The landing stage (right before viral penetration): ~104-nm spherical viral particle landed on the cell surface and formed a concave surface by wrapping ~1/4 of the virus surface (Fig. 4a). Between the virus and cell surface boundaries, there appeared with a concave-shaped hair-pin structure with a thickness of ~6 nm with two legs, ~67 and ~40 nm in length (indicated by two cyan arrows in Fig. 4a). The second object shows a quatrefoil-shaped structure ~21 nm in size (indicated by a yellow arrow in Fig. 4a). These two objects may represent two types of cell receptors that accumulate between viral and cell surfaces in response to viral binding through protein-protein interactions. Moreover, some spike-like particles (diameter of ~7 nm, black arrows indicated in Fig. 4a) were observed departing from the viral surface and merging into nanoparticles nearby (diameter of ~50 nm). We believed the ~7 nm spike-like particles represent to the viral glycoproteins such as glycoprotein VSV-G. Considering the interaction between HIV-1 spike protein and cell receptors triggered the exposure of a high-affinity binding site for a coreceptor,^57^ followed by the initiation of the membrane fusion process, the observed concave-shaped hair-pin structure and quatrefoil-shaped structure represent to the cell receptors and co-receptors.

To confirm above observations, the 3D map of this virus was reconstructed by IPET. IPET 3D projection and its superimposed 3D density map (a combination of positive and negative isosurface maps) confirmed that the spherical virus-like particle had spike-like proteins on its surface (Fig. 4a, Extended Data Fig. 4, and Supplementary Video 3). The accumulated densities observed within the concave surface of the cell may be due to cell receptor binding (hairpin-shaped density indicated by cyan arrows in Fig. 4a) with viral glycoprotein (white dots on virus surface) and the triggered downstream binding of proteins, such as the quatrefoil-shaped cell receptor (indicated by orange arrow in Fig. 4a). Moreover, next to the virus, the cell membrane displayed an abnormally flat, smooth and thick surface (indicated by pink arrows in Fig. 4a), which may indicate the formation of lipid rafts involved in viral entry, as reported.^58^

ii) In the penetrating stage, a globular viral particle with a diameter of ~130-nm half embeds into the cell membrane (Fig. 4b, Extended Data Fig. 5 and Supplementary Video S3). The half of the virus outside the cell membrane showed a smooth surface absent with coated spike-like particles. In contrast, the viral surface exhibited three attached nanoparticles with diameters of ~47 nm, ~64 nm and ~65 nm (black arrow in Fig. 4b), while these nanoparticles exhibited spike-like particles (similar size and shape), suggesting that the spikes were transferred from the virus to the nanoparticles. For the half of the virus embedded in the cell membrane, both the viral and cell membranes disappeared, which made it difficult to identify the boundary between virus and cell. Considering that these disappeared membranes are relatively large in size (the area is equal to three times the cross-sectional area of the virus), these membranes should have merged with the cell membrane and increased the local surface area of the cell based on the conventional mechanism of the nonendocytic route of viral fusion. In this mechanism, the viral membrane with its extra unbound spike proteins merged into the cell membrane and became a part of the cell membrane.^1^ However, considering that lipid rafts are also involved in virus entry^34,35^ via localization of cell receptor for HIV-1 entry,^59^ the accumulation of lipid rafts surrounding the virus should reduce the local mobility of the cell membrane, which should dramatically change the local landscape of the cell membrane after the great increase in its area by merging with the viral membrane. Changes in the landscape of the local cell membrane should be easily observed. However, while we did not observe significant changes in the cell membrane, such as wrinkles or distortions, we did observe three attached ~40-60-nm nanoparticles. Moreover, these extra unbound spikes from the merged viral membrane should preserve their biological functions in binding to nearby cell receptors on the targeted cell surface or the nearby cell surface. As a result, the cell surface morphology should be further modified by increasing the connectivity among the soft-irregular-shaped cell membrane boundaries or increasing the interactions and connectivity among the cells. However, the absence of observation of the accumulation of lipids or any change in cell surface morphology challenges the conventional mechanism of viral-cell membrane fusion.

One hypothesis to explain the above phenomena is that the extra membrane and unbound spike proteins formed attached ~50-nm nanoparticles surrounding the viral entry area. Based on the viral surface area calculation, the extra membrane from an embedded virus with a diameter of 109 nm (the peak population of the virus, shown in Fig. 3d) can produce ~2.9 particles with a diameter of ~55 nm (the secondary peak population of the surrounding particles, shown in Fig. 3d). The match between the calculation and our observation of the above three nearby nanoparticles (with diameters of ~45-65 nm) supports our hypothesis.

Additionally, in the EM images, the disappearance of the lipid membrane made it difficult to identify the boundary between the virus and cell. However, because of the uniform density of the virus core and its embedded half surrounded by tiny particles (~3-4 nm in diameter, white arrow indicated in Fig. 4b), the hemispherical shape of the virus embedded in the cell membrane was outlined, suggesting that the virus remained spherical after penetrating the cell.

iii) In the embedded stage, a virus particle with a diameter of ~96 nm was embedded underneath the cell membrane (Fig. 4c, Extended Data Fig. 6 and Video S3). The globular-shaped virus was surrounded by a layer of tiny particles with diameters of ~5 nm (arrows indicated in Fig. 4c). Considering i) the ~50 nm nanoparticles observed surrounding the cell (black arrows indicated in Fig. 4ab), ii) the lack of observation of the significant wrinkles or distortion of the local membrane (Fig. 4a), and iii) the lack of observation of the unbound spike-like proteins on the cell surface (Fig. 4a), these phenomena are consistent with our hypothesis, *i.e.*, the viral membrane was unwrapped from the virus surface with unbound spike-like proteins, formed into ~50-nm lipid-protein nanoparticles, and released into the surrounding solution. Underneath HIV surface membrane envelope, a layer of matrix protein p17 encapsulates a cone-shaped nucleocapsid that is formed by a layer of nucleocapsid protein p24 ^60,61^. Inside the capsid, the viral RNA is condensed with ribonucleoproteins and enzymes^62,63^. Remarkably, we found that this embedded virus remained in the form of a spherical shell of matrix proteins (Fig. 4b and c) instead of a cone-shaped viral capsid ^64^. This result adds another detail to the conventional model, in which the matrix protein shell underneath the viral membrane continues to protect the cone-shaped viral capsid from exposure to the cytoplasmic solution during the process of membrane fusion.

To further confirm the shape of the virus in the endosome, we examined two endocytosed particles (Fig. 4d and e, Extended Data Fig. 7 and 8 and Supplementary Video 3) respectively. One endocytosed particle has a globular virus-like (in diameter of ~62 x ~74 nm, indicated by the white arrows in Fig. 4d) adhered to a polygon-shaped density (in size of ~30 × ~45 nm, indicated by the orange arrows in Fig. 4d). The other endocytosed particle has a polygon-shaped density (in size of ~45 × ~50 nm) (Fig. 4b). Considering that these HIV capsids are in cone or polygon shapes instead of spheres,^65^ the observed polygon-shaped densities should be the capsid. The location of the capsid was observed inside the cell instead of near the boundary of the cell membrane, which suggests that the viral capsid is released inside the cell rather than near the cell membrane boundary during membrane fusion, consistent with our hypothesis.

### The hypothesis of the viral entry mechanism

Considering that the three lipid-spike-formed nanoparticles attached to the viral surface have diameters of ~47- ~65 nm (black arrow indicated in Fig. 4b), we asked whether the similarly sized nanoparticles that formed a majority in the extracellular solution (with a major peak of size population at ~55 nm, shown in Fig. 3b and c) also contained spike-like proteins. We zoomed into the structures of two extracellular particles (Fig. 4f and g, Extended Data Fig. 9 and 10, and Supplementary Video 3). One extracellular particle contains two adhered nanoparticles with diameters of ~50 nm and ~60 nm attached to the cell surface (Fig. 4f), while the other particle also contains a pair of adhered nanoparticles in diameter of ~50 nm and ~60 nm but not attached to the cell surface (Fig. 4g). Both of their 3D reconstructions showed that all these nanoparticles contained ~7 nm spike-like protein particles (indicated by the cyan color in Fig. 4f and g) based on their similar image contrasts and particle sizes to those inside the 3D maps of the virus-attached nanoparticles (black arrows indicated in Fig. 4b). These tests suggested that the ~50 nm extracellular nanoparticles are similar to the virus-attached nanoparticles, and both contain spike-like proteins, supporting our hypothesis.

## Discussion

**Figure 5:**
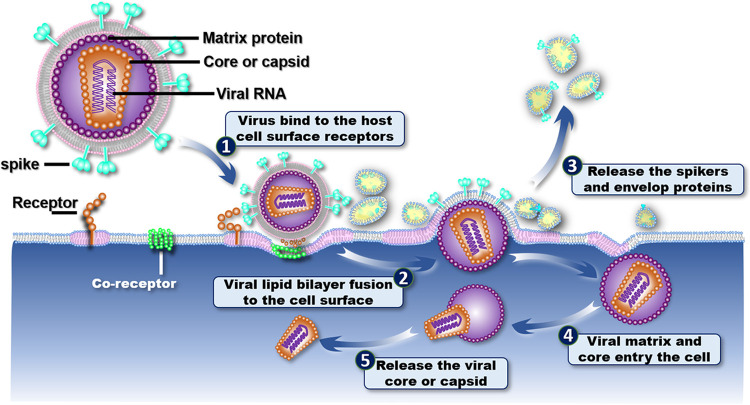
Schematics of the mechanism hypothesis of HIV entry into a cell. HIV consists of a lipid bilayer of viral membrane imbedded with surface spike protein trimers and a shell of matrix protein underneath the membrane, which surrounds a polygon or cone-shaped capsid with the viral RNA inside. To begin viral entry, the interaction between the viral spike proteins (cyan color) and the cell receptors on a lipid raft (pink color) triggers additional receptors to accumulate at the interface between the virus and cell membranes to make the cell membrane adopt a concave shape. Upon the viral membrane opening a pore, the virus begins to penetrate the cell membrane by squeezing the viral membrane and unbounded spikes to form ~50-nm protein-lipid nanoparticles under the association of lipid rafts. While the proteinlipid nanoparticles are released to the extracellular solution, the matrix proteins remain in their assembly in a spherical shell during the penetration of the cell membrane. The matrix protein shell is then gradually dissolved in the endosome to release its containing capsid deep inside the cell.

In this work, we have found a model for a time series of the viral entry process was retroactively produced by rearranging images of virus particles based on their distances from the cell membrane (Fig. 5) as follows. i) As the viral particles begin approaching the cell surface, spike protein gp120 on the virus surface interacts with the cell-surface receptor CD4 surrounded by a lipid raft, resulting in adherence. ii) The cell forms a concave surface that partially envelops the virus surface, and other cell receptors, such as CCR5 or CXCR4, are then triggered to accumulate at the interface, forming a bridge between the virus and cell membranes. iii) The viral membrane opens a pore by gradually squeezing the viral membrane away under spatial constraints from the lipid raft. The squeezed viral membrane and the unbound spike protein are wrapped together into three ~50-nm protein-lipid nanoparticles and released into the surrounding extracellular solution. iv) During viral penetration into the cell membrane, the shell formed by the matrix proteins underneath the viral membrane remains spherical in shape. v) After entering the cytoplasm, the shell of matrix proteins begins to disassemble before releasing the containing capsid to the cytoplasm. This model modified the conventional mechanism and provided additional details on the process of viral entry, *i.e.*, the unbound spike protein and the viral membrane is released into the extracellular solution instead of merging into the cell membrane, and the matrix-protein shell remains spherical in shape while the virus penetrates the cell membrane. These newly discovered details need to be validated by future biochemistry experiments or confirmed by further high-resolution 3D reconstructions.

In summary, we used the recently-developed liquid-phase TEM and IPET techniques to examine viral entry into HeLa cells in a wet environment at room temperature. The strength of this method in comparison to traditional methods includes that the whole-cell sample in its growth buffer can be examined without need for staining, drying, freezing, or slicing. This design allows us to visualize the intermediate stages of membrane fusion processes of viral entry. The observations contributed additional details to the conventional mechanism: for example, the viral lipid, the fact that the spike proteins were released from the viral surface and formed ~50 nm protein-lipid nanoparticles in the extracellular solution instead of merging with the cell membrane, and the spherical shell formed by the matrix proteins underneath the virus envelope maintaining its shape to protect the containing capsid during viral penetration. This singlevirus 3D imaging technique provides a new method for finding new antiviral drugs in the future.

## Methods

### Biological sample preparation

The HIV was the third-generation virus that was packaged with three co-transfection vectors, plasmids pLKO.1, psPAX2, and pMD2.G, in which pMD2.G contained genes for envelope proteins–the G glycoprotein of vesicular stomatitis Indiana virus (VSVG), which allow the infection of a broad range of cells, including HeLa cell ^66^. HeLa cells were grown in Gibco minimum essential media (MEM, containing 10% FBS) at 37 °C with 5% CO_2_, and virus-infected cells were prepared by incubating HeLa cells with virus at a ratio of 50:1 for 12 hours. The control GroEL used for negative staining imaging was provided by Dr. Scott Stagg’s laboratory: the protein at a concentration of ~0.5 mg/mL in Tris-buffered saline (TBS, 50 mM Tris, pH 7.4, 50 mM KCl, and 1 mM DTT) was diluted and stained by following the reported optimized negative-staining (OpNS) protocol^67^and then imaged by a Zeiss Libra 120 TEM (Carl Zeiss NTS) using a Gatan UltraScan 4k x 4k CCD. The cryo-EM images of GroEL were taken with an FEI Tecnai F20 G2 TEM using a Tietz 4k x 4k CCD camera at a defocus of −2.5 micrometers at 50,000x magnification (copyright belongs to Vossman, CC BY-SA 4.0, Wikimedia Commons).

### Liquid-phase TEM specimen preparation

The method to sandwich the biological samples follows the protocol that has been used to examine the real-time dynamic structures of liquid-vapor interfaces of liquid sodium.^42^ In brief, ~0.2 μl of sample solution (native liquid state, label-free, without any staining) was deposited and then sandwiched between two layers of 300-mesh TEM Formvar-coated copper grids (~3 mm in diameter, ~100 μm in window diameter) at room temperature with a humidity level of ~80%. The sandwiched grids were compressed together under a pressure of ~8 psi (~0.55 bar) for ~20 seconds. Any excess solution was then removed by filter paper before the edge of the sandwiched grids was sealed with vacuum grease and then mounted on a regular TEM holder.

### Optimizing TEM imaging

The liquid-phase TEM grids containing label-free biological samples were examined at room temperature using a Zeiss Libra 120 Plus TEM (Carl Zeiss NTS) operated at a high tension of 120 kV with 20 eV incolumn energy filtering. Micrographs were acquired on a Gatan UltraScan 4k × 4k CCD using a defocus up to 12 μm and magnification from 1,000× to 80,000× (each pixel of the micrographs corresponded to 107 Å to 1.48 Å, respectively, in the specimen). The radiation damage on the GroEL sample was tested using an illumination dose in the range from ~1 to ~36 e^−^/Å^2^ under magnification of 40,000× 80,000× and defocus up to −3 μm. Approximately 300 micrographs were acquired under low-dose conditions, a magnification of 80,000x, and defocus of ~0.1 – ~1.0 μm. Approximately 100 micrographs of HeLa cells were acquired under low-dose conditions at a magnification of 1,000× – 20,000×.

### Electron tomography (ET) tilt-series data acquisition

The liquid-phase EM holder for imaging HeLa cells was tilted at angles ranging from −36° to +60° in steps of 1° controlled by both Gatan tomography software and fully mechanically controlled automated electron tomography software,^68^ which were preinstalled in the microscope (Zeiss Libra 120 TEM). The TEM was operated at 120 kV with a 20 e^−^V energy filter. The tilt series were acquired by a Gatan Ultrascan 4k × 4k CCD camera with a defocus of ~11 μm and a magnification of 1,000× and 10,000× (corresponding to 107 Å and 10.7 Å per pixel, respectively, in the specimen). The total illumination electron doses were ~30 e^−^/Å^2^.

### Image preprocessing

The defocus and astigmatism of each untilled micrograph were measured, and the contrast transfer function (CTF) of each image was corrected using EMAN *CTfit* software.^69^ Prior to CTF correction, X-ray speckles were removed, and micrographs with distinguishable drift effects were excluded. To remove the strong background and enhance high-resolution details, each micrograph was filtered using a Gaussian boundary high-pass filter (at a resolution range of 200 nm to 500 nm). Tilt series of micrographs were initially aligned together with the IMOD software package.^70^ The CTF was then corrected by TOMOCTF.^71^ The tilt series of virus-like particles in square windows of 128 to 360 pixels (~137 to ~386 nm) were semi automatically tracked and windowed by IPET software.^9^

### Statistical analysis of virus-like particle sizes

For statistical analysis of particle sizes, all particles surrounding the cell (~315 in total) were selected and windowed. Each particle’s area was measured by Python Open CV.^72^ The histograms of the particles with diameters above and below 90 nm were fitted by Gaussian curves. The curves were merged based on the weights according to their particle numbers using a Python code.

### IPET 3D reconstruction

The tilt series of the targeted particles, including HeLa cells, viruses and nanoparticles, was reconstructed by IPET software.^9^ In brief, a circular mask with a Gaussian edge was applied to each image, followed by 3D reconstruction via an iteration refinement process with a series of soft-boundary masks and filters. To display the objects with positive or negative contrast, a superimposed 3D density map was generated by combining the positive contour map of the final IPET 3D density maps with its negative contour map by using Chimera software.^73^ The resolution of the final 3D map was estimated based on the intra-Fourier shell correlation (FSC).^9^ Briefly, the aligned images were then split into two groups based on having an odd- or even-numbered tilting index so that two 3D reconstructions were generated to compute the FSC curves, and the frequency at which the FSC curve fell to a value of 0.5 was used to represent the resolution.

## Supplementary Material

Supplement 1

Supplement 2

Supplement 3
